# Agreement between pulse oximetry and arterial oxygen saturation measurement in critical care patients during COVID-19: a cross-sectional study

**DOI:** 10.1007/s10877-022-00959-2

**Published:** 2023-01-21

**Authors:** Arthur James, Matthieu Petit, Flore Biancale, Adrien Bougle, Vincent Degos, Antoine Monsel, Antoine Vieillard-Baron, Jean-Michel Constantin

**Affiliations:** 1 Department of Anaesthesiology and Critical Care, Pitié-Salpêtrière Hospital, Sorbonne University, GRC 29, DMU DREAM, AP-HP, 75013 Paris, France; 2 Intensive Care Unit CHU Ambroise Paré, Université Paris-Saclay, UMR 1018, CESP, Villejuif, Boulogne, France

**Keywords:** Pulse oximetry, Hypoxia, Oxygen saturation, Intensive care unit

## Abstract

Some publications suggest that pulse oximetry measurement (SpO_2_) might overestimate arterial oxygen saturation (SaO_2_) measurement in COVID-19 patients. This study aims to evaluate the agreement between SpO_2_ and SaO_2_ among COVID-19 and non-COVID-19 patients. We conducted a multicenter, prospective study including consecutive intensive care patients from October 15, 2020, to March 4, 2021, and compared for each measurement the difference between SpO_2_ and SaO_2_, also called the systematic bias. The primary endpoint was the agreement between SpO_2_ and SaO_2_ measured with the Lin concordance coefficient and illustrated using the Bland and Altman method. Factors associated with systematic bias were then identified using a generalised estimating equation. The study included 105 patients, 66 COVID-19 positive and 39 COVID-19 negative, allowing for 1539 measurements. The median age was 66 [57; 72] years with median SOFA and SAPSII scores of, respectively, 4 [3; 6] and 37 [31; 47]. The median SpO_2_ and SaO_2_ among all measurements was respectively 97 [96–99] and 94 [92–96] with a systematic bias of 0.80 [− 0.6; 2.4]. This difference was, respectively, 0.80 [− 0.7; 2.5] and 0.90 [− 0.3; 2.0] among COVID-19 positive and negative patients. Overall agreement measured with the Lin correlation coefficient was 0.65 [0.63; 0.68] with 0.61 [0.57; 0.64] and 0.53 [0.45; 0.60] among the COVID-19 positive and negative groups, respectively. Factors independently associated with the variation of the SpO_2_-SaO_2_ difference were the PaO_2_/FiO_2_ ratio and need for mechanical ventilation. In our population, agreement between SpO_2_ and SaO_2_ is acceptable. During the COVID-19 pandemic, SaO_2_ remains an efficient monitoring tool to characterise the level of hypoxemia and follow therapeutic interventions. As is already known about general intensive care unit patients, the greater hypoxemia, the weaker the correlation between SpO_2_ and SaO_2_.

**Supplementary Information:**

The online version contains supplementary material available at 10.1007/s10877-022-00959-2.

## Introduction

Sars-Cov-2 infection, discovered in December 2019 in Wuhan, China, is responsible for severe cases of hypoxemia and for the development of acute respiratory distress syndromes (ARDS) [1–4].

Oxygenation during ARDS is one of the most important parameters to monitor. It can be done non-invasively by pulse oximetry to measure peripheral oxygen saturation (SpO_2_), which reflects the arterial oxygen saturation (SaO_2_), or invasively by arterial blood sampling to measure arterial oxygen pressure (PaO_2_). PaO_2_ is related to SaO_2_ by the haemoglobin dissociation curve with a sigmoid relation [5], and the ratio of PaO_2_ to the inspired fraction of oxygen (FiO_2_), also named PaO_2_/FiO_2_, is widely used to report the pulmonary capacity to oxygenate blood and therefore tissues [6]. A less invasive and simpler way of monitoring oxygenation capacity is to monitor SpO_2_ [7–9], which is particularly useful in guiding daily care as well as in judging the need for therapeutic interventions such as oxygen titration or monitoring in critical and acute situations. SpO_2_/FiO_2_ is, similarly to PaO_2_/FiO_2_, used to classify patients in studies [10–13] when PaO_2_ is not available.

Numerous studies have evaluated the agreement between SpO_2_ and SaO_2_ [14–16]. A strong correlation is reported with a bias between − 0.70 to + 1.86% [16]. Many factors have been reported to decrease the reliability of this measurement, such as hypoxemia, anaemia, hyperbilirubinaemia, skin pigmentation, low flow states or hypothermia [17–20]. Since the onset of the COVID-19 pandemic, some doubts have been raised about the quality of the agreement between SpO_2_ and SaO_2_ among COVID-19 patients. Wilson-Baig et al. published a retrospective, monocentric study of 17 intensive care unit patients and documented an average underestimation of the SaO_2_ by the SpO_2_ of 5.3% [21]. These findings triggered the hypothesis that a COVID-19 infection could influence the agreement between SaO_2_ and SpO_2_ through mechanisms including microvascular complications or variations in plasma protein levels, such as D-dimer, interleukin or ferritin, and that interacting with haemoglobin could change its spectral properties between 660 and 940 nm [22]. Nevertheless, the lack of a control group in the Wilson-Baig study limited the significance of its findings. Because SpO2 is a key component in the management of acute respiratory failure worldwide, especially in times of crisis and in emerging countries, we therefore conducted a study to investigate SpO2-SaO2 concordance in consecutive ICU patients, including COVID-19 positive and negative patients.

We thus conducted a study that aimed to investigate SpO_2_-SaO_2_ agreement among consecutive intensive care unit patients, including COVID-19 positive and negative patients.

## Materials and methods

### Study population

We conducted a prospective multicentric, observational study, including consecutive patients admitted in intensive care units (ICUs) with both COVID-19 and non-COVID-19 patients. Patients were admitted to five ICUs located in two different centres: four ICUs in La Pitié-Salpêtrière Hospital (Paris, France) and one ICU in Ambroise-Paré Hospital (Boulogne-Billancourt, France). All patients admitted in the five ICUs were consecutively included between October 15, 2020, and March 4, 2021, without exclusion criteria. The study was approved by the ethics committee of the French Society of Anaesthesiology and Intensive Care (IRB number 00010254-2021-103). Accordingly, with the European General Data Protection Regulation, we obtained an agreement of Assistance Publique des Hôpitaux de Paris for this project (ref. 20210525192353). This report follows the Strengthening the Reporting of OBservational studies in Epidemiology (STROBE) guidelines (Supplementary material 1) [23].

### Endpoints

The primary endpoint was the agreement between SpO_2_ and SaO_2_, analysed according to the method of Bland and Altman [24]. We defined the systematic bias of this agreement as the difference between SpO_2_ and SaO_2_.

Secondary endpoints were the concordance between SpO_2_ and SaO_2_ and the identification of factors independently associated with systematic bias.

### Data collection

This study used only routinely collected data. All existing pairs of SpO_2_ and SaO_2_ measurements were collected during the ICU stay within a limit of 30 days after ICU admission. The arterial sampling used to measure SaO_2_ was performed either by direct arterial puncture or by sampling from an arterial catheter line located in the radial or femoral position. The samples were analysed using a blood gas analyser (ABL90 FLEX, Radiometer, Denmark) and recorded. Just before each arterial sampling, the exact time was assessed and SpO_2_ was collected from the monitor. We thus used electronic medical record that report SpO_2_ min by min to confirm the measurement done at bedside. SpO_2_ measurement were done with the type of sensor available at the bedside (Supplementary material 2).

Concomitantly with SaO_2_ measurement, each arterial sample was used to measure PaO_2_, PaCO_2_, pH and lactate. At the time of each SaO_2_-SpO_2_ pair measurement, we also collected FiO_2_, temperature, haemoglobin and haematocrit, type of ventilation/oxygenation and patient position (supine or prone). The use of vasopressor, nitric oxide or corticosteroid therapy was also reported. Some biological parameters were collected when available, such as ferritin, D-dimer, fibrinogen, bilirubin and C-reactive protein.

### Statistical analysis

We considered the SaO_2_ to be the reference measurement. The systematic bias (defined as the SpO_2_-SaO_2_ difference) was described using the Bland and Altman method and reported with a mean measurement. Precision was reported with a 95% Bland–Altman Limit Of Agreement (LOA) [24]. The Bland–Altman method has been reported to sometimes provide biased estimates especially when the variances of the measurement errors of the two methods are different [25–27]. For this reason, we carried out the Taffé method using R statistical software package “*MethodCompare*” [28].

We described the relation between SpO_2_ and SaO_2_ using the Lin correlation coefficient for repeated data [29] and used a random-effect model (GEE [generalised estimating equation] type) to estimate the effect of different confounding factors on systematic bias while accounting that repeated measurements were made for several patients [30, 31]. Candidates’ variables included in the GEE model were selected when p-values were lower than 0.1 in univariate analysis and if considered important by the authors (AJ, JMC) according to their clinical expertise. We then conducted a stepwise backward regression and retained factors independently associated with a p-value lower than 0.05. Continuous variables were described using the median and the first and third quartiles, while categorical variables were described using relative number and percentage. We did not impute missing data except for missing haemoglobin at admission (n = 6), for which the mean between the previous and the next measurement was imputed. If those measurement was not available, if the missing data was the first of the series for example, we imputed the missing data with the closest in time. Data were analysed using R v4.0.2 (R-project.org, the R foundation for statistical Computing, Vienna, Austria).

## Results

### Patient characteristics

Between October 15, 2020, and March 4, 2021, 105 patients were consecutively included in the study. Among these patients, 66 were COVID-19 positive, representing 1238 blood samples with a median number of 11 [3, 23] samples per patient. Thirty-nine patients were COVID-19 negative, representing 301 samples with a median number of 5 [2, 11] samples per patient.

Patients’ age was 66 years [57, 72], with 79% being men, and length of ICU stay was 19 days [8, 39]. SOFA and SAPS2 were, respectively, 4 [3, 6] and 37 [31, 47]. Most patients included required mechanical ventilation (n = 74, 71.4%) or high-flow nasal canula oxygen therapy (n = 28, 26.7%). At ICU admission, PaO_2_/FiO_2_ was 142 [104, 248] with respectively 306 [225, 376] among COVID-19 negative patients and 119 [86, 141] among COVID-19 positive patients. COVID-19 positive patients experienced a period between symptom onset and ICU admission of 10 days [7, 13]. Admission causes for COVID-19 negative patients were general ICU (n = 17, 43.6%), neuro ICU (n = 13, 33.3%) and following cardiac surgery (n = 9, 23.1%). Overall, in-ICU observed mortality was 26.9% (n = 28), with 25.6% (n = 10) and 27.7% (n = 18) among COVID-19 negative and positive patients, respectively. Patients’ characteristics are reported in Table 1.

**Table 1:** Description of the population at the time of inclusion

Characteristics	NA	All patients (n = 105)	COVID-19 negative (n = 39)	COVID-19 positive (n = 66)	p-value
Sampling analysis					
Sample count		1539	301	1238	
Samples per patient	0	8 [2, 16]	5 [2, 11]	11 [3, 23]	0.013
Time since the onset of COVID-19 symptoms	39	10 [7, 13]	–	10 [7, 13]	–
Demographic description					
Age	0	66 [57, 72]	64 [51, 73]	67 [57, 71]	0.297
Male	0	83 (79.0)	29 (74.4)	54 (81.8)	0.466
Motif admission					< 0.001
– Cardiac surgery	0	9 (8.6)	9 (23.1)	0 (0.0)	
– COVID-19		66 (62.9)	0 (0.0)	66 (100.0)	
– Neurologic causes		13 (12.4)	13 (33.3)	0 (0.0)	
– Other		17 (16.2)	17 (43.6)	0 (0.0)	
Type of ventilation	0				< 0.001
– HFNC oxygen therapy		28 (26.7)	1 (2.6)	27 (40.9)	
– Mechanical ventilation		74 (71.4)	36 (94.9)	38 (57.6)	
–Spontaneous		2 (1.9)	1 (2.6)	1 (1.5)	
Severity					
ICU length of stay	0	19 [8, 39]	179 [6, 44]	19 [9, 36]	0.892
SAPS2	8	37 [31, 47]	47 [31, 56]	36 [31, 43]	0.051
SOFA	13	4 [3, 6]	7 [5, 11]	4 [3, 5]	< 0.001
Prone position requirement	0	9 (8.6)	1 (2.0)	8 (12.1)	0.184
Norepinephrine requirement	0	36 (34.3)	16 (41.0)	20 (30.3)	0.365
ICU mortality	1	28 (26.9)	10 (25.6)	18 (27.7)	1.000
Blood gas related parameters					
SpO_2_ (%)	0	96 [93, 98]	98 [96, 100]	94 [92, 96]	< 0.001
SaO_2_ (%)	0	96 [92, 98]	98 [96, 99]	94 [91, 96]	< 0.001
SpO_2_-SaO_2_	0	0.20 [-1.10, 1.40]	0.30 [-0.80, 1.00]	0.00 [-2.10, 1.50]	0.365
FiO_2_ (%)	0	55 [40, 75]	32 [24, 50]	62 [50, 80]	< 0.001
PaO_2_ (mmHg)	0	79 [66, 103]	99 [79, 135]	73 [64, 86]	< 0.001
PaO_2_/FiO_2_	0	142 [104, 248]	306 [225, 376]	119 [86, 141]	< 0.001
PaCO_2_ (mmHg)	0	39 [36, 44]	39 [35, 45]	39 [35, 44]	0.942
pH	0	7.43 [7.36, 7.47]	7.38 [7.33, 7.44]	7.45 [7.41, 7.47]	< 0.001
Lactates (mmol/L)	0	1.3 [0.8, 2.2]	0.9 [0.8, 1.6]	1.5 [0.9, 2.2]	0.018
Temperature (°C)	0	36.7 [36.2, 37.4]	36.5 [36.0, 37.2]	36.7 [36.3, 37.5]	0.064
Haemoglobin (g/dL)	7	12.0 [10.1, 13.2]	10.3 [9.2, 12.0]	12.6 [11.4, 13.8]	< 0.001
Other biological parameters					
Haematocrit (%)	32	36.8 [31.3, 39.5]	33.7 [27.9, 37.2]	37.0 [32.6, 39.9]	0.046
D-dimer (µg/L)	70	1260 [455, 2345]	–	1260 [455, 2345]	–
Fibrinogen (g/L)	42	6.7 [5.0, 7.4]	2.9 [2.4, 4.7]	6.8 [5.5, 7.5]	< 0.001
Bilirubin (mg/L)	42	8 [6, 15]	16 [6, 22]	8 [6, 14]	0.215
Ferritin (µg/L)	94	1391 [855, 1863]	–	1390 [1390, 1390]	–
CRP (mg/L)	97	106 [51, 203]	–	106 [51, 203]	–

*CRP* C-Reactive Protein; *FiO*_*2*_ inspired oxygen fraction; *HFO* High-Flow Nasal Canula Oxygen therapy; *ICU* intensive care unit; *NA* not available; *PaCO*_*2*_ partial pressure of arterial carbon dioxide; *PaO*_*2*_ partial pressure of oxygen in the arterial blood; *SOFA* sepsis-related organ failure assessment; *SaO*_2_ arterial oxygen saturation; *SpO*_2_, pulse oximetry measurement; *SAPS2* simplified acute physiology score 2. Variables are reported as median [IQR1, IQR3] or n (%). When a variable was not available at the time of admission, the closest measurement was used

In this study, 1239 blood gazes were matched with relevant clinical information measured at the time of the sampling. Among these samples, 301 were collected from COVID-19 negative patients while 1238 were collected from COVID-19 positive patients. From these 1539 blood samples, the mean PaO_2_/FiO_2_ was 250 [188–330] and 130 [90–178], respectively, for COVID-19 negative and positive patients. Information at the blood sample level is reported in Supplementary material 3.

### Primary endpoint

**Fig. 1:**
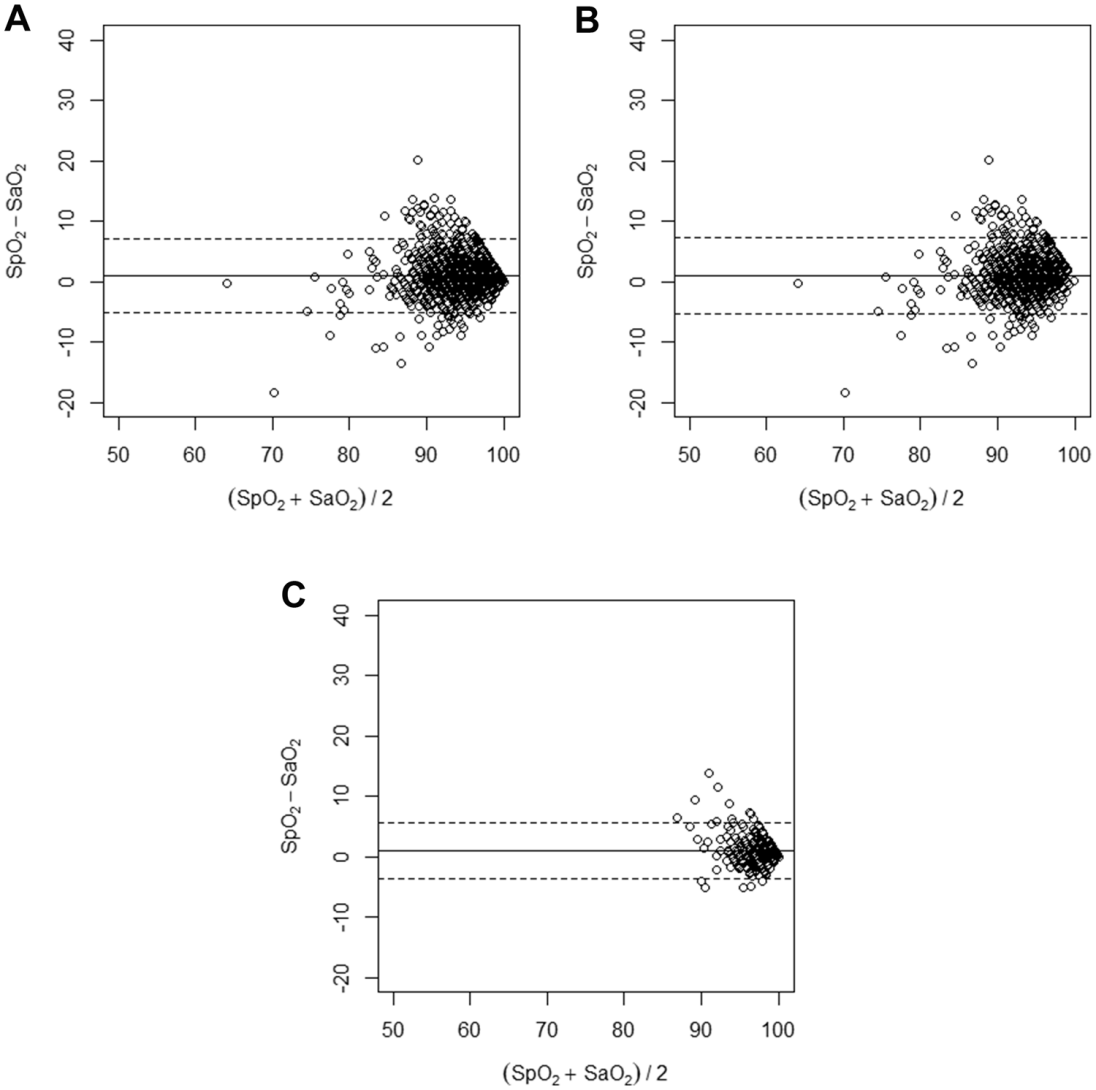
Bland and Altman analysis with continuous line being the systematic bias defined as SpO_2_-SaO_2_ and the dot line being the 95% limit of agreement of the systematic bias. **A** Included population. **B** COVID-19 positive patients. **C** COVID-19 negative patients

The mean systematic bias was of 1.0% among all included patients with a _95%_LOA [− 5.0; 7.1], highlighting that SpO_2_ overestimated SaO_2_ by 1.0% (Fig. 1A). The subgroup analysis identified a mean systematic bias of respectively 1.0% _95%_LOA [− 5.3; 7.4] (Fig. 1B) and 1.0% _95%_LOA [− 3.6; 5.6] (Fig. 1C) for COVID and non-COVID patients, respectively. These findings with the median (Q1-Q3) and the mean (95% CI) are summarised in Table 2. We report in Supplementary Material 4 the Bland–Altman plot resulting from the Taffé et al. approach and conducted to address the differences in variances between the measurement errors of the SpO_2_ and the SaO_2_.

**Table 2:** Description of the systematic bias defined as SpO_2_-SaO_2_ among all measurements

SpO_2_-SaO_2_	All included (n = 1,539)	COVID-19 negative (n = 301)	COVID-19 positive (n = 1,238)
Mean (Sd)	1.0 (3.1)	1.0 (2.3)	1.0 (3.2)
Mean (95% Bland–Altman LOA)	1.0 (− 5.0; 7.1)	1.0 (− 3.6; 5.6)	1.0 (− 5.3; 7.4)
Mean (95% CI)	1.0 (0.9; 1.1)	1.0 (0.8; 1.2)	1.0 (0.9; 1.1)
Median (Q1–Q3)	0.8 (− 0.6; 2.4)	0.9 (− 0.3; 2.0)	0.8 (− 0.7; 2.5)

*SpO*_*2*_ pulse oximetry measurement; *SaO*_*2*_ arterial oxygen saturation; *Sd* standard deviation; *CI* confidence interval; *LOA* Limit of Agreement

### Secondary endpoints

The Lin concordance coefficient between SpO_2_ and SaO_2_ was positive with r = 0.65 95% CI [0.63; 0.68]). Subgroup analysis highlighted a minimal between-group difference with r = 0.61 95% CI [0.57; 0.64] among COVID-19 positive patients and r = 0.53 95% CI [0.45; 0.60] among COVID-19 negative patients. A correlation plot is presented in Supplementary material 5.

Among the candidate variables, we retained eight variables after univariate analysis: COVID-19 status, baseline haemoglobin, PaO_2_/FiO_2_, mechanical ventilation requirements, prone position, noradrenaline infusion, PaO_2_ and temperature at the time of blood sampling (Supplementary material 6). The multivariate analysis identified the PaO_2_/FiO_2_ (p < 0.01, OR 1.0 95% CI [0.99; 1.0]) and mechanical ventilation requirement (p < 0.01, OR 5.5 95% CI [2.3; 13.3] as independently associated with an increase of systematic bias and not COVID-19 status (p = 0.37) (Table 3). Among COVID-19 patients, these two variables were also independently associated with an increase in systematic bias (p < 0.01, Supplementary material 7).

**Table 3:** Multivariate analysis among all measurements

	Intercept	p-value		OR
		Univariate analysis	Multivariate analysis	
COVID-19 positive	0.102	0.765	0.38	0.66 [0.32–1.53]
Haemoglobin	− 0.107	0.113	–	–
PaO_2_/FiO_2_	− 0.004	< 0.001	0.002	1.00 [0.99–1.00]
Mechanical ventilation requirement	1.556	< 0.001	< 0.001	5.52 [2.29–13.33]
Prone position requirement	0.279	0.160	–	–
Norepinephrine requirement	0.181	0.524	–	–
PaCO_2_	0.055	0.028	–	–
Temperature	0.006	0.940	–	–

*PaO*_*2*_ partial pressure of oxygen in the arterial blood; *PaCO*_*2*_ partial pressure of arterial carbon dioxide; *FiO*_*2*_ inspired oxygen fraction. For the multivariate analysis, we imputed missing data for the haemoglobin value (n = 6) with the average haemoglobin measured on the previous and following samples

The time since the first measurement had a statistically significant (p < 0.01) though clinically non relevant impact on the systematic bias with an increase of 0.05% for each supplementary day (Supplementary Material 8).

## Discussion

This study confirms that SpO_2_ and SaO_2_ measured among critical care patients have a good and clinically relevant agreement with a systematic bias of 1.00 _95%_ LOA [− 5.0; 7.1]. This agreement is also good for both COVID-19 negative and positive patient with systematic biases of, respectively, 1.0 _95%_LOA [− 3.6; 5.6] and 1.0 _95%_LOA [− 3.6; 5.6]. Nevertheless, the LOA distribution implies that there is uncertainty in the measurement of the SpO_2_ for example, a SpO_2_ of 90% could actually be either 85% or 97%. In multivariate analysis, more hypoxemic patients are more likely to have an increased systematic bias, while COVID-19 status is not associated with systematic bias variations. COVID-19 positive and negative patients have similar concordance coefficients between SpO_2_ and SaO_2_ as evaluated with the Lin coefficient with respective r coefficients of 0.61 and 0.53.

Contradictory results have been published concerning the agreement between SpO_2_ and SaO_2_ among COVID-19 patients. With on one side, two studies reporting the COVID-19 infection to be associated with a lower agreement between SpO_2_ and SaO_2_ [21, 32]. These studies were both retrospective, including a limited number of patients and without control groups. They mainly relied on two hypotheses. The first was that the major inflammatory state of COVID-19 patients requiring ICU care sustained a significant increase in blood concentration of proteins such as D-dimer or ferritin. It has been suspected that such inflammatory proteins could have absorption properties affecting the pulse oximeter signal, consequently reducing measurement accuracy [22]. The other hypothesis, supported by non-peer-reviewed publications, is that the virus would directly bind to haemoglobin and modify infrared absorption [33]. Our prospective study, with a larger cohort of patients and including a control group, did not confirm these preliminary results. We suggest that differences observed between SaO_2_ and SpO_2_ might be explained by the severity of hypoxemia rather than by COVID-19. On the other side, a recent study reported, among COVID-19 patients, a 0.05% bias with a limit of agreement ranging from − 2.2 to 2.3 which is consistent with the results presented in our study even if we report a wider limit of agreement [14].

The impact of hypoxemia on the measure of SpO_2_ is a well-known issue [34]. It has been reported that the poor quality of the measure of hypoxemic patients could be related 1/to the arteriolar dilation caused by tissue hypoxia that induces venous pulsation, which reduces the quality of the measure [9, 35], 2/with the quality of the pulse oximeter being used [35, 36] or high levels of skin pigmentation [14, 37, 38].

Several studies have reported low agreement between SpO_2_ and SaO_2_ among ICU patients [15, 20, 35, 39] with contradicting results: some report that SpO_2_ overestimates SaO_2_, and others report the opposite. In our study, we observed that SpO_2_ minimally overestimated SaO_2_ with a mean systematic bias of 1.0% _95%_LOA [− 5.0; 7.1].

This LOA spread implies that even with a good agreement and concordance with SaO_2_, SpO_2_ remains a daily routine monitoring device that needs to be confirmed by a SaO_2_ measurement whenever needed and especially when a precise measurement is required. This issues has been known for a long time [34] and a study, conducted in 2001, reported similarly a 2.1 standard deviation of the mean difference between SpO_2_ and SaO_2_. This study also reports that the type of oximeters, the presence of hypoxemia and the requirement for vasoactive drugs might influence SpO_2_ measurements [15]. To address this uncertainty, this study suggests that a SpO_2_ above 94% is necessary to ensure a SaO_2_ superior to 90%.

Our study has limitations. First, we conducted an observational study including COVID-19 positive and negative patients with significant differences both in baseline characteristics and outcomes, especially regarding hypoxemia. To account for these differences and allow for meaningful between-group comparisons, we proposed a multivariate analysis accounting for repeated measurements. One solution to address this issue would have been to include more hypoxemic and severely ill patients in the COVID-19 negative groups—those with ARDS, for instance.

Second, this study did not allow for an exploration of the hypothesis that inflammatory biological markers could influence systematic bias. These markers, such as D-dimer, fibrinogen, bilirubin, ferritin or C-reactive protein, were indeed inconstantly measured among COVID-19 positive patients and rarely measured among COVID-19 negative patients. Other factors, such as skin colour, methemoglobinemia, carboxyhaemoglobinia or hyperbilirubinemia, were also not considered.

Third, while invasive mechanical ventilation allows for accurate FiO2 measurement, other modes of oxygenation may not allow for such accuracy. In a modeling article, Wagstaff et al. report that HFO is the only oxygenation method to guarantee a given FiO2, whereas for other oxygenation methods FiO2 is not stable. Indeed, FiO2 decreases when the respiratory rate increases [40]. Indeed, FiO2 decreases when the respiratory rate increases.

Finally, we did not analyse the impact of the type of pulse oximeters used. Devices are known to cause substantial differences in bias and precision, especially at low saturation [34] and the COVID-19 surge led hundreds of manufacturers proposing new devices with sometime questionable standard quality. This reinforces the importance for caregivers to ensure a careful attention the devices selected in their wards especially when caring for hypoxemic patients.

## Conclusion

This study confirms existing data about the reliability of SpO_2_ measurement in ICUs and suggests that possible differences between the COVID-19 positive and negative populations might be related to the higher prevalence of severe hypoxemia among COVID-19 positive patients’. In clinical practice, it remains important to acknowledge that SpO_2_ measurement reliability worsens with hypoxemia severity. Further studies are needed to explore the potential impact, if any, of inflammatory proteins on the accuracy of SpO_2_.

## Supplementary Information

Below is the link to the electronic supplementary material.

Supplementary file1 (DOCX 190 KB)

## Data Availability

Relevant data can be available after reasonable request to the corresponding author and discussion with the working group.
