# Supplementary material for: Agreement between pulse oximetry and arterial oxygen saturation measurement in critical care patients during COVID-19: a cross-sectional study
Source: J Clin Monit Comput. 2023 Jan 21;37(4):977–84. doi: 10.1007/s10877-022-00959-2 (PMC9859741; doi:10.1007/s10877-022-00959-2)
Supplement: Supplementary file 1 — Supplementary file1 (DOCX 190 KB) [file 10877_2022_959_MOESM1_ESM.docx]

# Supplementary materials

## Supplementary material 1: STROBE checklist

|  | Item No | Recommendation | Page |
| --- | --- | --- | --- |
| Title and abstract | 1 | (*a*) Indicate the study’s design with a commonly used term in the title or the abstract | 1 |
|  |  | (*b*) Provide in the abstract an informative and balanced summary of what was done and what was found | 1 |
| Introduction | | |  |
| Background/rationale | 2 | Explain the scientific background and rationale for the investigation being reported | 4 |
| Objectives | 3 | State specific objectives, including any prespecified hypotheses | 4 |
| Methods | | |  |
| Study design | 4 | Present key elements of study design early in the paper | 5 |
| Setting | 5 | Describe the setting, locations, and relevant dates, including periods of recruitment, exposure, follow-up and data collection | 5 |
| Participants | 6 | (*a*) Give the eligibility criteria and the sources and methods of selection of participants. Describe methods of follow-up | 5 |
|  |  | (*b*) For matched studies, give matching criteria and number of exposed and unexposed | - |
| Variables | 7 | Clearly define all outcomes, exposures, predictors, potential confounders and effect modifiers. Give diagnostic criteria, if applicable | 5-6 |
| Data sources/ measurement | 8* | For each variable of interest, give sources of data and details of methods of assessment (measurement). Describe comparability of assessment methods if there is more than one group | 5-6 |
| Bias | 9 | Describe any efforts to address potential sources of bias | 5 |
| Study size | 10 | Explain how the study size was arrived at | 5 |
| Quantitative variables | 11 | Explain how quantitative variables were handled in the analyses. If applicable, describe which groupings were chosen and why | - |
| Statistical methods | 12 | (*a*) Describe all statistical methods, including those used to control for confounding | 6 |
|  |  | (*b*) Describe any methods used to examine subgroups and interactions | 6 |
|  |  | (*c*) Explain how missing data were addressed | 6 |
|  |  | (*d*) If applicable, explain how loss to follow-up was addressed | - |
|  |  | (*e*) Describe any sensitivity analyses | 6 |
| Results | | |  |
| Participants | 13* | (a) Report numbers of individuals at each stage of study—e.g., numbers potentially eligible, examined for eligibility, confirmed eligible, included in the study, completing follow-up, and analysed | 7-8 |
|  |  | (b) Give reasons for non-participation at each stage | - |
|  |  | (c) Consider use of a flow diagram | - |
| Descriptive data | 14* | (a) Give characteristics of study participants (e.g., demographic, clinical, social) and information on exposures and potential confounders | 7 |
|  |  | (b) Indicate number of participants with missing data for each variable of interest | 8 |
|  |  | (c) Summarise follow-up time (e.g., average and total amount) | 8 |
| Outcome data | 15* | Report numbers of outcome events or summary measures over time | - |
| Main results | 16 | (*a*) Give unadjusted estimates and, if applicable, confounder-adjusted estimates and their precision (e.g., 95% confidence interval). Make clear which confounders were adjusted for and why they were included | 10 |
|  |  | (*b*) Report category boundaries when continuous variables were categorised | - |
|  |  | (*c*) If relevant, consider translating estimates of relative risk into absolute risk for a meaningful time period | - |
| Other analyses | 17 | Report other analyses done—e.g., analyses of subgroups and interactions, and sensitivity analyses | 11-12 |
| Discussion | | |  |
| Key results | 18 | Summarise key results with reference to study objectives | 15 |
| Limitations | 19 | Discuss limitations of the study, taking into account sources of potential bias or imprecision. Discuss both direction and magnitude of any potential bias | 15 |
| Interpretation | 20 | Give a cautious overall interpretation of results considering objectives, limitations, multiplicity of analyses, results from similar studies and other relevant evidence | 15 |
| Generalisability | 21 | Discuss the generalisability (external validity) of the study results | 15-16 |
| Other information | | |  |
| Funding | 22 | Give the source of funding and the role of the funders for the present study and, if applicable, for the original study on which the present article is based | 18 |

## Supplementary material 2: Pulse oximeter references

Reusable:

- Finger oximeters: 512E, Mindray,
- Hear oximeters: ES – 2414-15, EnviteC

Non-reusable:

- Fingers oximeters: DA-22-11-1, EnviteC

## Supplementary material 3: Patient baseline characteristics at the moment of each measurement

|  | **COVID-19 negative**  **n = 301** | **COVID-19 positive**  **n = 1,238** |
| --- | --- | --- |
| **SpO_2_ (%)** | 98 [96 - 99] | 95 [92 - 97] |
| **SaO_2_ (%)** | 97 [96 – 99] | 94 [91 - 96] |
| **Systematic bias** | 0,9 [-0,30 - 2] | 0,8 [-0,7 - 2,5] |
| **FiO_2_ (%)** | 40 [30 - 50] | 60 [45 - 70] |
| **PaO_2_** | 88 [77 - 107] | 73 [64 - 87] |
| **PaCO_2_** | 39 [35 - 43] | 44 [39 - 49] |
| **PaO_2_/FiO_2_** | 250 [188 - 330] | 130 [90 - 178] |
| **pH** | 7,40 [7,35 - 7,45] | 7,44 [7,38 - 7,47] |
| **Haemoglobin (g/dL)** | 9,9 [9 - 11,1] | 10,3 [8,8 - 11,9] |
| **Temperature (°C)** | 37 [36,5 - 37,5] | 36,9 [36,4 - 37,5] |
| **Blood lactates (mmol/L)** | 0,9 [0,7 - 1,3] | 1,3 [0,9 - 1,7] |
| **Prone position (%)** | 3 (1) | 166 (13,5) |
| **Norepinephrine (%)** | 152 (50.6) | 375 (30,5) |
| **Ventilation mode (%)**   - HFNC Oxygen thereapy - Mechanical - Spontaneous | 8 (2,7)  290 (96,3)  3 (1) | 144 (11,6)  1090 (88)  4 ( 0,3) |
| **Steroids use (%)** | 9 (45) | 413 (68,0) |
| **d-dimer (µg/L)** | NA | 1225 [695 - 2275] |
| **Fibrinogen (g/L)** | 4,3 [2,7 - 5,2] | 7 [5,7 - 7,9] |

SpO_2_, pulse oximetry measurement; SaO_2_, arterial oxygen saturation; ICU, intensive care unit; PaO_2_, partial pressure of oxygen in the arterial blood; PaCO_2_, partial pressure of arterial carbon dioxide; SAPS2, simplified acute physiology score 2 ; SOFA, sepsis-related organ failure assessment; FiO_2_, inspired oxygen fraction; HFNC Oxygen therapy, High-Flow Nasal Canula Oxygen therapy; NA, not available. Variables are reported as median [IQR1, IQR3] or n (%). When a variable was not available at the time of admission, the closest measurement was used.

## Supplementary Material 4: Bland Altman plot resulting from the Taffé et al. approach


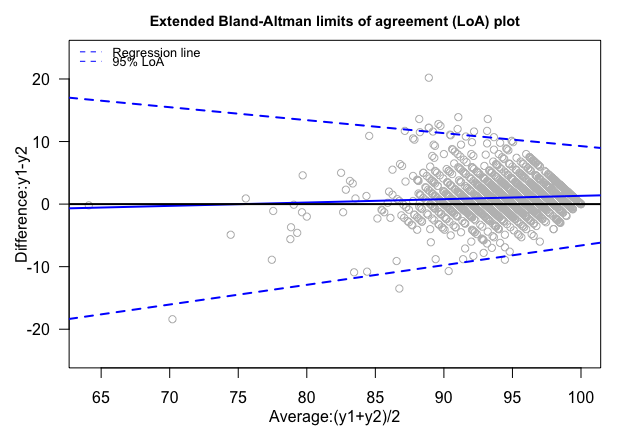


## Supplementary material 5: Correlation plot for COVID-19 positive and negative patients


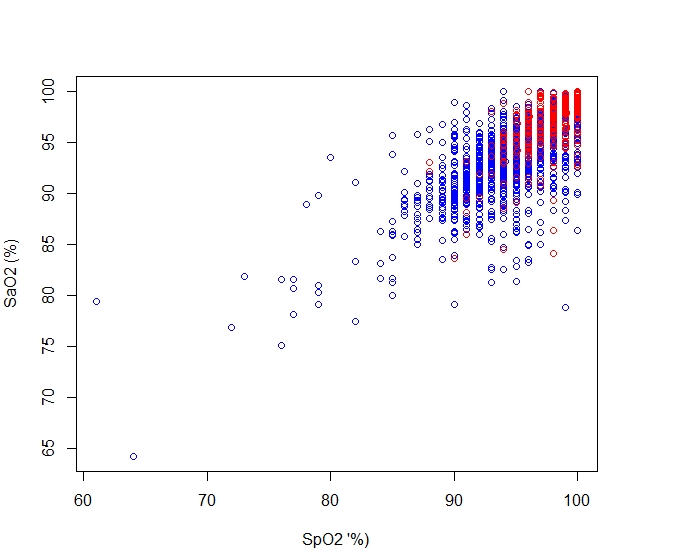


SpO_2_, pulse oximetry measurement; SaO_2_, arterial oxygen saturation; red dots are COVID-19 negative patients; blue dots are COVID-19 positive patients.

## Supplementary material 6: Univariate analysis

|  | **Systematic bias ≤2** | **Systematic bias >2** | **p-value** |
| --- | --- | --- | --- |
| **n** | 1103 | 436 |  |
| **COVID-19 positive** | 872 (79.1) | 366 (83.9) | 0.035 |
| **SpO_2_** | 95 [92, 97] | 97 [95, 99] | <0.001 |
| **SaO_2_** | 95 [93, 97] | 93 [90, 95] | <0.001 |
| **PaO_2_/FiO_2_** | 147 [105, 227] | 140 [107, 194] | 0.053 |
| **pH** | 7.43 [7.38, 7.47] | 7.43 [7.36, 7.47] | 0.266 |
| **PaCO_2_** | 42 [37, 47] | 44 [39, 49] | <0.001 |
| **Haemoglobin (g/dL)** | 10.6 [9.2, 12.1] | 9.5 [8.5, 11.5] | <0.001 |
| **Prone position requirement** | 112 (10.2) | 57 (13.1) | 0.119 |
| **Norepinephrine requirement** | 371 (33.6) | 156 (35.8) | 0.460 |
| **Temperature** | 36.9 [36.4, 37.5] | 36.9 [36.4, 37.5] | 0.923 |
| **Blood lactates** | 1.2 [0.9, 1.7] | 1.2 [0.8, 1.7] | 0.572 |
| **Mechanical ventilation requirement** | 971 (88.0) | 490 (93.8) | <0.001 |

SpO_2_, pulse oximetry measurement; SaO_2_, arterial oxygen saturation; PaO_2_, partial pressure of oxygen in the arterial blood; PaCO_2_, partial pressure of arterial carbon dioxide; FiO_2_, inspired oxygen fraction. Variables are reported as median [IQR1, IQR3] or n (%). When a variable was not available at the time of admission, the closest measurement was used.

## Supplementary material 7: Multivariate analysis among COVID-19 positive patients (n=1,238 samplings)

|  | **Intercept** | **p** | **P final** |
| --- | --- | --- | --- |
| **PaO_2_/FiO_2_** | -0.005 | 0.100 | 0.056 |
| **Mechanical ventilation requirement** | 2.217 | <0.001 | <0.001 |
| **Prone position** | 0.418 | 0.667 | - |
| **norepinephrine requirement** | -0.067 | 0.917 | - |
| **PaCO_2_** | 0.016 | 0.586 | - |
| **Temperature** | 0.072 | 0.482 | - |

PaO_2_, partial pressure of oxygen in the arterial blood; PaCO_2_, partial pressure of arterial carbon dioxide; FiO_2_, inspired oxygen fraction. Variables are reported as median [IQR1, IQR3] or n (%). When a variable was not available at the time of admission the closest measurement was used.

*Supplementary material 8: Relation between the time elapsed since the first measurement and the systematic bias*

*
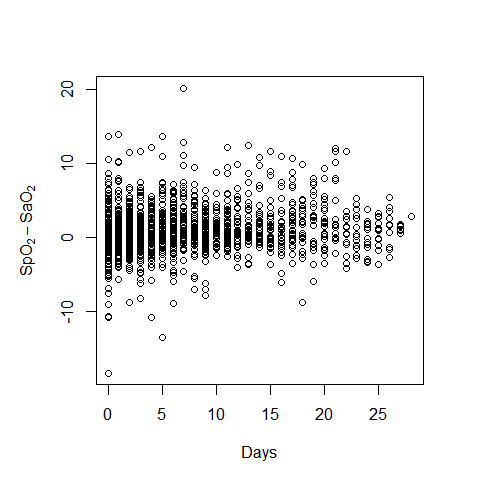
*
